# Circulating Linoleic Acid is Associated with Improved Glucose Tolerance in Women after Gestational Diabetes

**DOI:** 10.3390/nu10111629

**Published:** 2018-11-02

**Authors:** Ulrika Andersson-Hall, Nils-Gunnar Carlsson, Ann-Sofie Sandberg, Agneta Holmäng

**Affiliations:** 1 Institute of Neuroscience and Physiology, Sahlgrenska Academy, University of Gothenburg, 405 30 Gothenburg, Sweden; agneta.holmang@gu.se; 2 Division of Food and Nutrition Science, Department of Biology and Biological Engineering, Chalmers University of Technology, 412 96 Gothenburg, Sweden; nils-gunnar.carlsson@chalmers.se (N.-G.C.); ann-sofie.sandberg@chalmers.se (A.-S.S.)

**Keywords:** gestational diabetes mellitus, serum fatty acids, linoleic acid, glucose tolerance

## Abstract

Women with previously diagnosed gestational diabetes mellitus (GDM) are at increased risk of type-2-diabetes mellitus (T2D). We aimed to establish links between glucose tolerance (GT) and serum fatty acid (FA) profile in the transition from GDM to T2D. Six years after GDM, 221 women were grouped as having normal GT (NGT), impaired GT (IGT), or T2D based on oral GT test results. Fasting serum FAs were profiled, anthropometric measures taken, and dietary intake determined. Linoleic acid (LA) was significantly higher in NGT women (*p* < 0.001) compared with IGT and T2D, and emerged as a strong predictor of low glucose and insulin levels, independently of BMI. Self-reported vegetable oil consumption correlated with LA serum levels and glucose levels. Delta-6-, delta-9-, and stearoyl-CoA-desaturase activities were associated with decreased GT, and delta-5-desaturase activities with increased GT. In a subgroup of women at high risk of diabetes, low LA and high palmitic acid levels were seen in those that developed T2D, with no differences in other FAs or metabolic measurements. Results suggest that proportions of LA and palmitic acid are of particular interest in the transition from GDM to T2D. Interconversions between individual FAs regulated by desaturases appear to be relevant to glucose metabolism.

## 1. Introduction

Gestational diabetes mellitus (GDM), defined as glucose intolerance with onset or first recognition during pregnancy, has short- and long-term implications for both mother and child [1,2]. In the mother, it confers increased risk of developing glucose intolerance later in life. In fact, the risk factors for GDM and T2D are the same, and it has been suggested that the inherent insulin-resistant state of pregnancy may reveal a pre-disposition for T2D. Even though normal glucose tolerance is typically resumed after birth, women with a prior diagnosis of GDM have a sevenfold increased risk of developing T2D later in life [3]. The risk increases over the first 5 years after giving birth, reaching a plateau after 10 years [4]. The years immediately after a GDM pregnancy, therefore, represent an important window for close health surveillance and intervention in order to prevent the development of T2D.

Fatty acids (FAs) are believed to play an important role in the progression of insulin resistance and T2D. Both the total amount of circulating FAs, and the relationship between different types of FAs, may impact on functions such as membrane fluidity, regulation of substrate oxidation, appetite receptor activation, inflammation, glucose transporter inhibition, lipotoxicity, and beta-cell function, which are all important in the development of metabolic disease [5,6,7,8]. In recent years, nutritional recommendations have shifted to focus more on fat quality than fat quantity, with observational studies showing that substituting dietary saturated FAs (SFAs) with mono- or poly-unsaturated FAs (MUFAs and PUFAs, respectively) is associated with improved insulin sensitivity [6,9].

Serum FA levels, which depend both on dietary intake and synthesis/degradation within the body, provide a more objective measure of FA levels than estimates based on dietary intake; some circulating FAs correlate well with dietary intake (e.g., linoleic acid (LA), eicosapentaenoic acid (EPA), and docosahexaenoic acid (DHA)), whereas others show weaker correlations with dietary measures (e.g., α-linolenic acid (ALA)) [10]. Relative serum levels of individual FAs also give information on the conversion rates between different FAs, and allow estimation of the activity of key enzymes such as the FA desaturases, which seem to play a role in the development of T2D [11].

Although there are limited studies on individual circulating FAs and T2D, it is clear that high serum levels of saturated FAs are generally associated with increased risk of T2D [12,13,14], whereas there have been mixed reports on the relationship between serum levels of individual MUFAs and PUFAs and T2D [15,16,17,18,19]. A majority of these studies to date have been conducted in elderly or middle-aged populations and so it is of interest to explore whether the same patterns hold true in younger populations, in this case a younger population of women with increased risk of T2D. Though a few reports exist on FAs during diabetic pregnancies [20,21,22], to our knowledge, only one prior study has analysed circulating levels of individual FAs after GDM [23]. This study was performed soon after birth, showed associations of specific FAs with overweight and adiposity, but failed to show a link to insulin resistance. Our aim is to use FA-profiling to explore associations between individual FAs/desaturase activities and glucose tolerance in women six years after GDM, in order to identify biomarkers that are related to development of impaired glucose tolerance and T2D.

## 2. Materials and Methods 

### 2.1. Subjects

The study was approved by the ethics committee at the University of Gothenburg (402-08/750-15). Informed consent was obtained from all participants. A group of 542 women in the Gothenburg area who were diagnosed with GDM from 2005 to 2009 were invited to participate in the study; 378 of this group were interviewed by telephone and asked to attend a follow-up visit. The study group included the 237 women who attended the follow up visit 5.6 ± 0.5 years after pregnancy, and has previously been described in detail [24]. 

### 2.2. Protocol

At follow-up visit, fasting blood samples were collected from all participants. Women who had not been diagnosed with diabetes since pregnancy also underwent a 2-h 75 g oral glucose tolerance test (OGTT). Venous blood for analysis of plasma glucose and serum insulin was collected at 0 (fasting value), 30, 60, 90, and 120 min. Fasting serum and plasma blood samples were used for further analysis.

During the visit, anthropometric variables (weight, height, waist and hip circumference) and resting blood pressure were measured, and participants were asked to complete dietary and lifestyle questionnaires, as previously described [24]. Dietary intake was assessed using a semi-quantitative food frequency questionnaire; the questionnaire has previously been validated in Swedish men and women against a 4-day food record and 24-h energy expenditure and nitrogen excretion [25]. Only correctly completed questionnaires were used for analysis. Basal metabolic rate (BMR) was calculated using the Henry equation [26]; subjects with an energy intake (EI)/BMR ratio <0.95 or >3.0 were excluded from energy and macronutrient analyses. Frequency of meals containing meat, fish, or vegetarian options was determined based on specific questions. Participants were further asked to estimate quantities of different cooking fats used, allowing assessment of the proportion of butter, margarine and vegetable oil within the diet. Educational level was based on the women’s highest attained level and classified as elementary school (level 0), 2 years of high school (level 1), 3 years of high school (level 2), <3 years of university (level 3) or ≥3 years of university (level 4). At a second follow-up visit, body composition was measured by BOD POD (software version 5.4.0, Cosmed, Rome, Italy) in a subgroup of 87 women. 

### 2.3. Glucose Tolerance Groups

Women were split into three groups based on findings at 6-year follow-up: normal glucose tolerance (NGT), IGT (including impaired fasting glucose or impaired glucose tolerance), and T2D (including subjects with previously diagnosed T2D), as previously described [24]. Classification of NGT, IGT, and T2D was based on the 1999 WHO guidelines [27]; NGT, fasting glucose < 6.1 mmol/L and 2-h glucose < 7.8 mmol/L; IGT, fasting glucose ≥ 6.1 and <7.0 mmol/L and/or 2-h glucose ≥ 7.8 and <11.1 mmol/L; T2D, fasting glucose ≥ 7.0 mmol/L or 2-h glucose ≥ 11.1 mmol/L. Individuals with confirmed type 1 diabetes mellitus at follow-up (*n* = 8) were excluded from the analysis. 

For the T2D group, diagnosis was on average 1.1 ± 1.6 years before the study visit (date of diagnosis ranged from 5 years before study visit to at study visit). There were no differences in levels of any FA studied between those with previously known (*n* = 21) and newly diagnosed T2D (*n* = 22); time of diagnosis was not associated with any FA measurements. Women with T2D were therefore treated as a single group.

### 2.4. Biochemical Measurements

HbA1c was analysed immediately with a point-of-care analyser (Afinion AS100; Axis-Shield, Oslo, Norway). Glucose, insulin, cholesterol (total, low-density lipoprotein (LDL), and high-density lipoprotein (HDL)), and triglycerides were analysed at the accredited Clinical Chemistry Laboratory, Sahlgrenska University Hospital (International Standard ISO 15189:2007). The homeostatic model assessment of insulin resistance (HOMA-IR) was calculated as (fasting glucose × fasting insulin)/22.5 [28]. 

### 2.5. Fatty Acid Analysis

Fasting serum samples for FA analysis were available from 221 women. Serum FA composition was determined in duplicate samples as FAME by direct methylation of 50 µL serum samples [29]. To each sample, was added 25 µg C17 (Larodan, Stockholm, Sweden) as internal standard, 1 mL Acetyl chloride 10% in Methanol, and 1 mL Toluene. Methylation occurred in tightly closed tubes placed in a 70 °C water bath for 2 h and shaken every 30 min. After cooling, 3 mL petroleum ether and 0.5 mL H_2_O were added, and the tubes shaken and centrifuged mildly (2500 rpm) for 5 min at room temperature. Then the upper phase was withdrawn and evaporated at 40 °C under a stream of N_2_, and the FA residue was dissolved in 200 µL iso-octane. The methylated samples were injected into an Agilent 7890 A GC system equipped with a VF-WAXms (30 m × 0.25 mm × 0.25 µm d_f_) column (Agilent J&W Scientific, Santa Clara, CA, USA) and interfaced with a Agilent 5975 C triple-axis mass spectrometric detector in electron-impact mode. Injection volume was 1 µL with a 15:1 split ratio at an inlet temperature of 275 °C. The carrier gas was Helium at a fixed flow of 1 mL/min. The temperature program was as follows: 100 °C for 0 min, increasing by 4 °C/min to 205 C, thereafter increasing at 1 °C/min up to 230 °C, followed by 5 min at 230 °C. External standard GLC-463 (Nu-Chec prep, Inc., Elysian, MN, USA) was used for identification and quantification. FA concentrations were calculated as percentages of total FAs, and desasturase activities were estimated by the ratios 20:3n6/18:2n6 (delta-6-desaturase, D6D), 20:4n6/20:3n6 (delta-5-desaturase, D5D), 16:1n7/16:0 (Stearoyl-CoA desaturase, SCD) and 18:1n9/18:0 (delta-9-desaturase, D9D). 

### 2.6. Statistical Analysis

For between-group comparisons, ANOVA and ANCOVA (with BMI as covariate and with the assumption of homogeneity of regression slopes fulfilled) were used with Tukey post-hoc analysis. Pearson bivariate and partial correlations (with BMI as covariate) were performed on overall data from all participants. Stepwise regression was performed for HOMA-IR with all FAs entered as independent variables. All analyses were conducted using IBM SPSS version 23.0 (IBM SPSS Statistics, Armonk, NY, USA). Values are expressed as mean ± SD. *P* < 0.05 was considered significant.

## 3. Results

### 3.1. Background Characteristics

At the 6-year follow-up visit, 135 women had NGT, 43 women had IGT, and 43 had T2D. Table 1 shows characteristics of the three groups. BMI, fat mass, and waist and hip circumference were significantly greater in women in the IGT and T2D groups compared with the NGT group. Women with IGT or T2D also had significantly lower HDL-cholesterol levels than women with NGT, and women with T2D had significantly higher triglyceride levels compared with those with NGT. Smoking rate and alcohol intake did not differ between groups. Fewer of the T2D group were of Scandinavian origin compared with the other two groups, and they had lower level of education. All glucose, HbA1c, and insulin measurements were higher in the IGT and T2D groups compared with the NGT group (Table 1). Based on self-reported dietary intake (Table 2), there were no differences between groups in energy intake, macronutrient intake, or specific meal frequencies. However, there were differences in the proportions of different cooking fats used between groups; women with T2D reported using a significantly greater proportion of margarine than women with NGT, while women with T2D or IGT reported using a significantly lower proportion of butter than women with NGT. 

### 3.2. Fatty Acid Profiles in NGT, IGT, and T2D Groups

Circulating FA profiles for the three groups are displayed in Table 3. Individual FAs are expressed as a percentage of total FAs. Between-group differences were seen for PUFAs both before and after adjustment for BMI; the amount of LA as a proportion of all FAs was significantly lower in women in the IGT and T2D groups than in the NGT group, and proportions of GLA and DGLA were significantly higher in women in the IGT group compared with those in the NGT group. The proportion of docosapentaenoic acid (DPA) was higher in T2D than both other groups only after BMI adjustment. For MUFAs, the proportion of palmitoleic acid (POA) was significantly higher in women in the IGT and T2D groups, and the proportion of oleic acid was significantly higher in women in the IGT group, compared with women with NGT. Only the proportion of POA for the IGT versus NGT group remained significant after adjustment for BMI. Among the saturated FAs, the only between-group difference in proportions was for palmitic acid which was significantly higher in the T2D versus the NGT group both before and after adjustment for BMI.

Delta-6 desaturase activity (D6D) was found to be higher in women in the IGT and T2D groups than in the NGT group, and stearoyl-CoA desaturase (SCD) activity was higher in the IGT compared with the NGT group. Differences remained after adjustment for BMI. There were no significant differences for delta-5 (D5D) or delta-9 desaturase.

### 3.3. Correlation between FAs and Clinical Measurements for All Women

DHA and LA correlated inversely with BMI (Table 4), whereas GLA, DGLA, POA, and oleic acid correlated positively with BMI. LA also correlated negatively with hip and waist circumference, blood pressure (systolic and diastolic), and % body fat, and DHA with waist and hip circumference. Positive correlations with anthropometric data were found for GLA, DGLA, POA, and oleic acid. Saturated myristic and palmitic acids correlated positively with waist circumference.

HDL-cholesterol correlated with many individual FAs; ALA, all MUFAs, and myristic and palmitic acids correlated negatively with HDL, whereas EPA, DPA, DHA, LA, AA, and stearic acid correlated positively with HDL. The opposite pattern was observed for triglycerides, with strong negative correlations with DHA, LA, AA, and stearic acid and positive correlations with ALA, all MUFAs, and myristic and palmitic acids. 

D6D, D9D, and SCD correlated positively, but D5D negatively, with BMI, waist and hip circumference, % body fat, and levels of LDL and triglycerides. The reverse was seen for HDL, which correlated negatively with D6D, D9D, and SCD, but positive with D5D. 

There were no correlations between individual FAs or desaturases and age (data not shown).

### 3.4. Correlation between FAs and Glucose Tolerance for All Women

Correlations between FAs and glucose and insulin measurements are shown in Table 5. Amongst the ω-3 PUFAs, ALA correlated positively with all glucose and insulin measurements, whereas DHA and DPA correlated negatively with AUC glucose and AUC insulin, respectively. For ω-6-PUFAs, LA correlated inversely with all glucose and insulin measurements and with HbA1c. Similarly, AA correlated inversely with fasting and AUC insulin and AUC glucose. DGLA, however, correlated positively with fasting and AUC insulin. MUFAs were associated with high glucose and insulin levels; POA correlated positively with AUC glucose and both fasting and AUC insulin, and oleic acid with both fasting and AUC, glucose, and insulin. Saturated myristic and palmitic acids both correlated positively with fasting insulin and AUC glucose and insulin. Palmitic acid correlated with fasting glucose and HbA1c. All correlations of FAs with glucose and insulin measurements described above remained significant after adjustment for BMI, whereas palmitic acid was the only variable that correlated with HbA1c after adjustment (data not shown).

For the desaturases, D6D, SCD and D9D were positively correlated, and D5D negatively correlated, with fasting insulin and both AUC glucose and insulin. Only D9D correlated with fasting glucose and HbA1c. All of these relationships remained significant after adjustment for BMI.

A stepwise regression analysis was performed to predict the variance of insulin resistance (HOMA-IR), using all fatty acids as independent variables. The final model resulted in an adjusted R^2^ = 0.23 and included three FAs; LA (β = −0.40, *p* < 0.001), ALA (β = 0.27, *p* < 0.001) and DPA (β = −0.17, *p* = 0.007).

**Figure 1:**
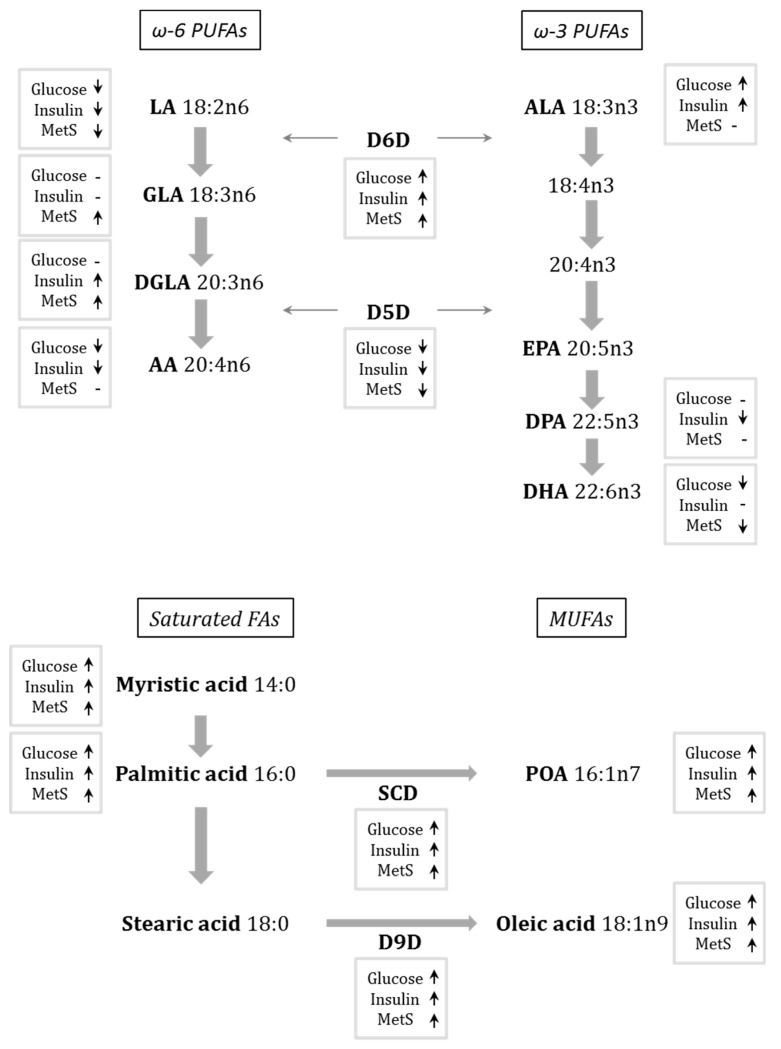
Summary of results from serum fatty acid (FA) profiling in women 6 years after GDM. Arrows shows whether the FA or desaturase had a positive (arrow up), or negative (arrow down) correlation with glucose values (fasting or AUC), with insulin (fasting or AUC) after BMI adjustment, or with at least two parameters of MetS (high BMI, low HDL cholesterol, high blood pressure, high waist circumference, or high body fat percentage). AA, arachidonic acid; ALA, α-linolenic acid; AUC, area under the curve; BMI, body mass index; D5D, delta-5 desaturase; D6D, delta-6 desaturase; D9D, delta-9 desaturase; DGLA, dihomo-γ-linolenic acid; DPA, docosapentaenoic acid; DHA, docosahexaenoic acid; EPA, eicosapentaenoic acid; GLA, γ-linolenic acid; HDL, high-density lipoprotein; LA, linoleic acid; MUFA, monounsaturated fatty acid; POA, palmitoleic acid; PUFA, polyunsaturated fatty acid; SCD, stearoyl-coenzyme A desaturase.

An overview of the associations of FAs and desaturases with glucose and insulin metabolism, and risk factors for metabolic syndrome, is presented in Figure 1. 

### 3.5. Correlations with Dietary Intake in All Women

Self-reported fish meal frequency correlated with EPA and DHA (r = 0.20, *p* = 0.01 for EPA; r = 0.30, *p* < 0.001 for DHA), and it also correlated with level of education (r = 0.17, *p* = 0.04). Meat meal frequency correlated negatively with ALA (r = −0.25, *p* = 0.001) and vegetarian meal frequency positively with stearic acid (r = 0.17, *p* = 0.03). Fish, meat, or vegetarian meal frequency, however, did not correlate with any anthropometric measurement, with lipid, glucose or insulin values, or with ethnicity (data not shown).

Proportions of butter, margarine, and vegetable oil used showed several correlations, both with serum FAs and with glucose tolerance and anthropometric data. Of special note were the positive correlation between vegetable oil intake and LA (r = 0.19, *p* = 0.03), and the inverse correlation between margarine intake and LA (r = −0.21, *p* = 0.02). The opposite pattern was seen for palmitic acid, which correlated negatively with vegetable oil (r = −0.23, *p* = 0.006) and positively with margarine intake (r = 0.18, *p* = 0.04). MUFAs generally correlated positively with margarine intake (combined association r = 0.26, *p* = 0.002). Furthermore, glucose AUC correlated positively with margarine intake (r = 0.21, *p* = 0.02), but negatively with both butter and oil intake (r = −0.18, *p* = 0.04 and r = −0.22, *p* = 0.02, respectively). For anthropometric and clinical measures, vegetable oil intake correlated favourably with several parameters, amongst which were negative correlations with systolic blood pressure (r = −0.27, *p*=0.002) and BMI (r = −0.22, *p* = 0.01), although a positive correlation was seen with fat free mass (r = 0.25, *p* = 0.05).

### 3.6. FA Influence on T2D Development in Women Treated with Insulin during Pregnancy

Considering women who received insulin treatment during pregnancy as at high-risk of developing T2D (*n* = 51), we evaluated which factors differed in those who developed (*n* = 21) or had not developed T2D (*n* = 30) by the 6-year follow-up (Table 6). Among the fatty acids, LA levels were significantly lower and palmitic acid levels significantly higher in women who had developed T2D compared with those who had not. No other FA or desaturase differed between groups (data not shown). There were also no between-group differences in terms of anthropometric data, lipid measurements, ethnicity, or dietary intake.

## 4. Discussion

In a population of women with previous GDM, distinct FA profiles were linked to glucose intolerance and insulin resistance 6 years after pregnancy. A high level of LA correlated with high consumption of vegetable oil and was consistently associated with a healthy metabolic profile, as well as high insulin sensitivity, glucose tolerance, and a low incidence of T2D. D6D activity correlated positively with measures of insulin resistance, and was increased in women with IGT and T2D, whereas D5D correlated negatively with insulin concentration, but activity levels did not differ significantly between groups. Results for ω-3 PUFAs were mixed, with only ALA correlating positively with insulin resistance. Levels of saturated fatty acids, the desaturases SCD and D9D, and corresponding MUFAs, correlated with margarine intake and were generally associated with decreased metabolic health and glucose intolerance. In women with most severe cases of GDM in which insulin treatment was needed during pregnancy, the development of T2D development was linked to high levels of palmitic acid and low levels of LA. Taken together, these findings highlight that LA concentrations after GDM may be a predictor of glucose tolerance and risk of developing T2D. 

### 4.1. MUFAs and Saturated FAs Associate with Decreased Metabolic Health 

Saturated FAs are high in the western-style diet and are linked to harmful metabolic profiles. The role of MUFAs in metabolic health are, however, not as clear [30,31]. In our study, serum levels of the MUFAs, POA and oleic acid, appear to be related to total body fat, whereas levels of saturated FAs correlated with visceral fat (waist) only and not hip or overall fat. POA, oleic acid, myristic acid, and palmitic acid were all associated with increased glucose intolerance and insulin resistance, and with high use of margarine for cooking. Both MUFAs and saturated FAs were strongly linked to lipoprotein and triglyceride levels. It should be noted that the concentration of these FAs not only depend on intake from fat but also from de-novo lipogenesis during excessive calorie intake from other energy sources, for example, if intake of refined sugars is high. We could not deduct from our dietary data the most likely origin of high serum levels of saturated FAs as we did not see correlations with fat intake or with mono- and disaccharide intake (data not shown). Furthermore, with margarine in Sweden being mainly plant based, correlations between saturated FAs and proportion of margarine use might be unexpected. There is, however, saturated fats such as palmitic acid present in plant oils and it is also worth noting that the FFQ question on margarine, vegetable oil, and butter use was expressed as proportions and not in absolute values.

In the current population, as in similar populations [32], the strongest predictor for T2D development after GDM was the requirement for insulin treatment during pregnancy, which predicted 80.5% of T2D cases at the 6-year follow up [24]. We therefore considered women with insulin treatment during pregnancy as a high risk group and looked at T2D development within this group. Although there were no differences in BMI, anthropometry, lipoprotein, triglyceride, or ethnicity between women, high levels of palmitic acid, together with low levels of LA, were characteristic of women who had developed T2D from within this high-risk group. There were no significant differences in dietary intake between groups in the high risk women (data not shown), though this might partially be explained by a low number of dietary questionnaires accepted after the exclusion criteria (*n* = 15 and *n* = 10 in groups NGT/IGT and T2D, respectively). 

The discrepancy between previous reports of the overwhelming benefits of oleic acid intake [33], and the detrimental associations between serum levels and diabetes risk [31,34], can partly be explained by the simultaneously high activities of desaturases. When saturated FA levels are high, desaturases convert these FAs to their unsaturated forms in order to reduce cell toxicity. D9D activity, converting stearic acid to oleic acid, correlates strongly both with anthropometric measures and glucose metabolism in our study, and could therefore be responsible for the production of high oleic acid levels seen with increased body fat and insulin resistance. Similarly, high levels of POA in insulin resistance could be explained by increased activity of SCD. It is also worth noting that in Northern and Western Europe, oleic acid intake is often derived from dairy and meat sources, and is therefore strongly linked to saturated FA intake [35], again pointing to the importance of studying both FA intake and circulating FA levels in different populations.

### 4.2. LA Is Robustly Associated with Healthy Glucose Homeostasis

LA is the most abundant of the PUFAs, and the decrease in LA levels in the T2D and IGT groups largely confirms results from previous studies [15,16,19,36]. LA was inversely associated with all anthropometric measures, and correlated strongly with both fasting and AUC, glucose and insulin, before and after adjustment for BMI. Furthermore, decreased LA was the strongest predictor of insulin resistance (HOMA-IR) in our regression model. LA was also low in women who developed T2D in our high-risk subgroup. 

Low LA levels are either a result of low dietary intake of LA or increased breakdown of LA (via D6D activity), or a combination of the two [13]. We saw a correlation between LA serum concentration and vegetable oil intake. But, we also found increased D6D activity to be associated with T2D for the GDM population as a whole, suggesting a combination of low LA intake and high breakdown. In our high-diabetes-risk population, however, there was no difference in D6D or in downstream FAs between those that developed T2D and those that did not, supporting the argument that intake of LA might be decisive. This is also supported by the finding of an inverse association between AUC glucose levels and vegetable oil intake in the whole population.

Inverse correlations of AA levels with glucose and insulin, in agreement with studies in other populations [37,38], further supports the link between high ω6-PUFA proportions and lower risk for insulin resistance. DGLA, however, correlated in a positive manner with insulin levels, both fasting and AUC. D6D converts LA to DGLA and has been suggested to be activated at high insulin levels [39], which could explain the association we see between high insulin levels and DGLA. As seen in other studies, DGLA was also associated quite strongly with BMI, waist circumference, and body fat [31,40]. 

### 4.3. ω3-PUFAs Show Mixed Results in Relation to Glucose Metabolism

As expected, we found that high fish intake led to higher serum levels of EPA and DHA. Although there was no difference in EPA or DHA between glucose tolerance groups, there was an inverse correlation between glucose AUC and DHA, and DHA in particular was associated with good metabolic health, as seen in correlations with anthropometric measured, HDL and triglycerides. Fish oil supplementation has been shown to be beneficial to metabolic health, and our results add to some previous evidence of an association between DHA, in particular, and increased insulin sensitivity, although results are not clear cut [9,41]. ALA has been studied less than DHA and EPA, but the few studies on serum ALA levels have shown either no association with glucose levels and T2D incidence [14,15], or in one case an inverse association with T2D [16]. Surprisingly, we found that high ALA levels correlated with high concentrations of both glucose and insulin. To our knowledge, such an association has not been shown before. The correlation with glucose, however, was only observed within the T2D group and not the NGT or IGT groups (data not shown). The mixed results in previous studies could therefore be explained by between-cohort differences in terms of glucose tolerance and diabetes progression. It is, however, worth noting that ALA is rapidly oxidized [42] and has a very low abundance in serum (less than 1% of total FAs - approximately 50 times lower concentration than, for example, LA). This may make correlation analysis and conclusions regarding ALA precarious.

A recent systematic review showed that the relationship between PUFA intake, individual FA serum levels and diabetes risk is linked to polymorphisms of the genes expressing D5D and D6D (*FADS1* and *FADS2*, respectively). High D5D activity and low D6D activity is suggested to play a protective role in the development of diabetes, where these activities are influenced both by FA intake and gene polymorphisms. For example, high intake of ω-3 PUFAs improved HOMA-IR for subjects with some *FA desaturate (FADS)* gene variants, whereas it increased fasting glucose for carriers of a minor *FADS2* allele [11].

### 4.4. Strengths and Limitations

Few studies have used an OGTT to evaluate glucose tolerance and insulin levels in relation to FA profile. Studies in the field have generally used fasting glucose or clinical diagnosis of T2D as outcome measures. In addition, previous research on individual FAs in relation to glucose metabolism has mainly been conducted in middle-aged or elderly populations with no identified underlying risk for T2D [14,15,16]. The women in our study are relatively young and have a sevenfold increased risk of developing T2D compared with women with no previous GDM [43]. The study includes a well-characterized cohort with a broad range of different levels of glucose tolerance. Use of a cohesive single-sex cohort with a narrow age span has the advantage that many variables are already controlled for, but means that there are limitations when extrapolating the results to an extended population. The study is cross-sectional with lipid profile measured at only one time point, making it difficult to determine causality. To evaluate risk of diabetes development further, data collection at additional time points would be desirable. There could potentially be genetic confounders affecting the relationship between FAs and glucose tolerance. We have chosen to adjust analysis for BMI but not for ethnicity or family history of diabetes since this data, collected from medical records, was uncertain for many women. However, if further adjusting for known heredity and ethnicity based on our available data, all significant BMI adjusted differences were still significant (data not shown). We have further chosen to measure total FA fractions in serum and previous results have indicated that results from different circulating fractions show good agreement [44]. Furthermore, in line with most other reports, we decided to express FAs as a percentage of total FAs rather than as absolute values. Using percentages of FAs also gives the advantage of relatively strong agreement between dietary intake and serum levels for LA, EPA, and DHA [10]. ALA serum levels, however, correlate weakly with dietary intake [10].

## 5. Conclusions

Given the high incidence of T2D in women with previous GDM, it is of great interest to find links between FA metabolism and glucose tolerance during the transition from GDM to T2D, both in order to predict disease progression and enable clinicians to give evidence-based advice. Our results show that proportions of serum LA and palmitic acid are particularly important predictors of diabetes risk, suggesting that an increase in dietary LA intake could be beneficial. Our finding that vegetable oil consumption correlated positively with LA serum levels and OGTT glucose levels in this group further supports this idea. The role of desaturase activities in the promotion of an optimal balance between different FAs which might influence the risk for developing T2D and IGT warrants further study.

## Figures and Tables

**Table 1:** Characteristics of women 6 years after a GDM pregnancy. Women were divided into glucose tolerance groups based on OGTT at 6-year visit; type 2 diabetes (T2D) was determined by OGTT at time of diagnosis or at 6-year visit.

		NGT			IGT			T2D		*P* ^a^		
	*n*	Mean	SD	*n*	Mean	SD	*n*	Mean	SD	IGT vs. NGT	T2D vs. NGT	T2D vs. IGT
Age (years)	135	39.5	5.0	43	40.5	6.0	43	37.8	5.3			0.05
Ethnicity (% Scandinavian)	130	53.1		43	51.2		43	32.6			0.023	
Education level (0–4)	125	2.4	1.3	41	2.3	1.3	37	1.6	1.5		0.007	
BMI (kg/m^2^)	135	26.1	4.9	43	29.5	4.6	43	29.2	5.8	<0.001	0.002	
Waist circumference (cm)	133	87.4	10.8	42	94.0	16.7	43	95.0	14.6	0.007	0.004	
Hip circumference (cm)	133	102.3	9.9	42	108.2	9.1	43	107.5	11.6	0.002	0.02	
Body fat (%)	59	32.9	7.9	16	40.5	6.6	12	38.5	13.2	0.007		
Fat mass (kg)	59	23.4	9.5	16	32.3	10.7	12	31.8	15.8	0.01	0.04	
Fat free mass (kg)	59	45.6	5.4	16	46.0	6.7	12	45.9	6.0			
BP systolic (mmHg)	134	114	13	43	123	14	43	119	14	<0.001		
BP diastolic (mmHg)	134	75	10	43	79	10	43	78	11			
s-Total cholesterol (mM)	135	4.7	0.8	43	4.7	0.8	42	4.7	0.7			
s-HDL (mM)	135	1.5	0.3	43	1.3	0.3	43	1.4	0.5	0.001	0.04	
s-LDL (mM)	135	2.9	0.8	43	3.2	0.7	43	3.0	0.6			
s-Triglycerides (mM)	134	0.9	0.5	43	1.2	0.5	43	1.4	1.4		<0.001	
Alcohol intake (g)	111	3.3	4.1	34	2.8	4.3	22	1.8	3.2			
Smokers (%)	126	19.8		37	13.5		34	11.8				
*Glucose and insulin*												
b-HbA1c (mmol/mol)	135	37.1	3.7	42	38.4	3.9	43	52.7	19.0		<0.001	<0.001
p-Glucose fasting (mM)	135	5.4	0.4	42	6.1	0.4	43	8.2	3.5	0.02	<0.001	<0.001
p-Glucose 2 h (mM)	125	5.5	1.1	41	7.7	1.6	12	11.8	3.9	<0.001	<0.001	<0.001
s-Insulin fasting (mU/L)	131	8.1	4.2	42	14.7	9.4	43	12.9	7.5	<0.001	<0.001	
s-Insulin 2 h (mU/L)	119	45.0	37.8	39	87.8	57.7	12	85.3	58.4	<0.001	0.008	
HOMA-IR	130	1.9	1.0	42	3.9	2.7	42	4.9	3.4	<0.001	<0.001	0.04

^a^ Between-group comparisons based on ANOVA. b, whole blood; BP, blood pressure; BMI, body mass index; GDM, gestational diabetes mellitus; HDL, high-density lipoprotein; HOMA-IR, homeostatic model assessment of insulin resistance; IGT, impaired glucose tolerance; LDL, low-density lipoprotein; NGT, normal glucose tolerance; p, plasma; s, serum; SD, standard deviation.

**Table 2:** Self-reported dietary intake of women 6 years after a GDM pregnancy. Women were divided into glucose tolerance groups based on OGTT at 6 year visit; type 2 diabetes (T2D) was determined by OGTT at time of diagnosis or at 6-year visit.

	NGT (*n* = 88)		IGT (*n* = 32)		T2D (*n* = 17)		*P* ^a^			*P*^a^ BMI-Adjusted		
	Mean	SD	Mean	SD	Mean	SD	IGT vs. NGT	T2D vs. NGT	T2D vs. IGT	IGT vs. NGT	T2D vs. NGT	T2D vs. IGT
Energy intake (kcal/d)	2399	551	2354	727	2248	590						
Carbohydrate intake (g/d)	251	66	251	87	232	73						
Protein intake (g/d)	97	23	98	38	97	27						
Fat intake (g/d)	108	32	103	33	101	30						
*Meal frequencies*												
Meat meals (per week)	7.8	3.5	7.9	2.3	7.3	3.2						
Fish meals (per week)	2.6	1.9	2.7	1.5	3.0	2.5						
Vegetarian meals (per week)	1.4	2.4	0.6	1.3	0.7	1.5						
*Proportion of cooking fat used*												
Butter (%)	32	31	17	25	12	20	0.03	0.03		0.01	0.01	
Margarine (%)	24	30	36	36	51	36		0.01			0.004	
Vegetable oil (%)	43	28	38	30	37	33						

^a^ Between-group comparisons based on ANOVA (P) and ANCOVA (BMI-adjusted P). BMI, body mass index; GDM, gestational diabetes mellitus; IGT, impaired glucose tolerance; NGT, normal glucose tolerance; SD, standard deviation.

**Table 3:** Serum fatty acid (FA) composition 6 years after GDM, expressed as % of total FAs. Women were divided in glucose tolerance groups based on OGTT at 6 year visit; type 2 diabetes (T2D) was determined by OGTT at time of diagnosis or at 6-year visit.

		NGT (*n* = 135)		IGT (*n* = 43)		T2D (*n* = 43)		*P* ^a^			*P*^a^ BMI-Adjusted		
		Mean	SD	Mean	SD	Mean	SD	NGT vs. IGT	NGT vs. T2D	IGT vs. T2D	NGT vs. IGT	NGT vs. T2D	IGT vs. T2D
*ω-3 PUFAs, %*													
α-Linolenic acid	ALA (18:3n3)	0.72	0.22	0.81	0.25	0.78	0.29						
Eicosapentaenoic acid	EPA (20:5n3)	0.90	0.54	1.00	0.67	0.95	0.45						
Docosapentaenoic acid	DPA (22:5n3)	0.41	0.09	0.40	0.08	0.45	0.12					0.007	0.036
Docosahexaenoic acid	DHA (22:6n3)	1.77	0.61	1.72	0.61	1.70	0.64						
*ω-6 PUFAs, %*													
Linoleic acid	LA (18:2n6)	30.1	3.8	27.6	3.8	27.1	4.2	0.001	<0.001		0.004	<0.001	
γ-Linolenic acid	GLA (18:3n6)	0.30	0.15	0.38	0.14	0.34	0.14	0.004			0.011		
Dihomo-γ-linolenic acid	DGLA (20:3n6)	1.25	0.31	1.42	0.34	1.36	0.40	0.013					
Arachidonic acid	AA (20:4n6)	6.06	1.36	6.09	1.09	6.00	1.34						
*MUFAs, %*													
Palmitoleic acid	POA (16:1n7)	1.42	0.50	1.77	0.70	1.68	0.80	0.004	0.042		0.026		
Vaccenic acid	(18:1n7)	2.03	0.29	2.06	0.30	2.08	0.33						
Oleic acid	(18:1n9)	22.6	2.6	23.9	3.1	23.7	3.8	0.03					
*Saturated FAs, %*													
Myristic acid	(14:0)	0.64	0.28	0.73	0.28	0.69	0.28						
Palmitic acid	(16:0)	23.5	2.1	23.9	2.0	24.7	2.3		0.002			0.003	
Stearic acid	(18:0)	8.41	0.94	8.32	0.77	8.49	1.24						
*Desaturase activity*													
Delta-6 desaturase	D6D (DGLA/LA)	0.047	0.015	0.058	0.016	0.056	0.017	<0.001	0.002		0.002	0.010	
Delta-5 desaturase	D5D (AA/DGLA)	5.3	1.9	4.7	1.7	4.8	1.7						
Stearoyl-CoA desaturase	SCD (16:1n7/16:0)	0.060	0.018	0.073	0.024	0.067	0.027	0.003			0.02		
Delta-9 desaturase	D9D (18:1n9/18:0)	2.8	0.5	2.9	0.5	2.8	0.6						

^a^ Between-group comparisons based on ANOVA (P) and ANCOVA (BMI-adjusted P). BMI, body mass index; CoA, coenzyme A; GDM, gestantional diabetes mellitus; IGT, impaired glucose tolerance; MUFA, monounsaturated fatty acid; NGT, normal glucose tolerance; PUFA, polyunsaturated fatty acid; SD, standard deviation.

**Table 4:** Correlations of serum fatty acids with anthropometric measurements and blood lipids for all women. R = Pearson correlation coefficient. Significant correlations (*p* < 0.05) are displayed in bold.

		BMI	Systolic BP	Diastolic BP	Waist	Hip	Body Fat %	HDL	LDL	TG
		*n* = 221	*n* = 220	*n* = 220	*n* = 218	*n* = 218	*n* = 85	*n* = 221	*n* = 221	*n* = 220
*ω-3 PUFAs*										
**ALA (18:3n3)**	R	0.109	−0.013	−0.033	0.114	0.072	0.120	−**0.325**	**0.170**	**0.385**
	P	0.106	0.850	0.629	0.092	0.291	0.275	**<0.001**	**0.011**	**<0.001**
**EPA (20:5n3)**	R	−0.045	0.075	0.062	−0.080	−0.075	−0.019	**0.161**	<0.001	−**0.179**
	P	0.506	0.269	0.356	0.239	0.270	0.860	**0.016**	0.998	**0.008**
**DPA (22:5n3)**	R	−0.101	−0.034	−0.022	−0.117	−0.098	−0.146	**0.161**	−0.043	−0.059
	P	0.134	0.620	0.743	0.085	0.149	0.181	**0.017**	0.527	0.386
**DHA (22:6n3)**	R	−**0.196**	−0.059	−0.040	−**0.180**	−**0.221**	−0.162	**0.306**	−0.126	−**0.275**
	P	**0.003**	0.384	0.556	**0.008**	**0.001**	0.138	**<0.001**	0.061	**<0.001**
*ω-6 PUFAs*										
**LA (18:2n6)**	R	**−0.265**	**−0.163**	**−0.184**	**−0.202**	**−0.184**	**−0.247**	**0.232**	−0.060	**−0.549**
	P	**<0.001**	**0.016**	**0.006**	**0.003**	**0.006**	**0.023**	**0.001**	0.376	**<0.001**
**GLA (18:3n6)**	R	**0.208**	0.065	0.094	0.075	**0.150**	**0.394**	−0.063	**0.208**	0.049
	P	**0.002**	0.334	0.166	0.273	**0.027**	**<0.001**	0.351	**0.002**	0.473
**DGLA (20:3n6)**	R	**0.223**	0.076	0.052	**0.209**	**0.216**	**0.289**	−0.080	**0.161**	−0.035
	P	**0.001**	0.261	0.447	**0.002**	**0.001**	**0.007**	0.235	**0.017**	0.603
**AA (20:4n6)**	R	−0.019	0.054	0.048	−0.092	−0.004	−0.127	**0.260**	−0.120	−**0.373**
	P	0.781	0.428	0.481	0.178	0.958	0.245	**<0.001**	0.076	**<0.001**
*MUFAs*										
**POA (16:1n7)**	R	**0.305**	0.125	**0.160**	**0.204**	**0.266**	**0.400**	−**0.176**	**0.206**	**0.511**
	P	**<0.001**	0.063	**0.017**	**0.002**	**<0.001**	**<0.001**	**0.009**	**0.002**	**<0.001**
**Vaccenic acid (18:1n7)**	R	0.104	0.034	0.078	0.009	0.085	0.078	−**0.207**	−0.008	**0.335**
	P	0.124	0.618	0.246	0.891	0.213	0.480	**0.002**	0.907	**<0.001**
**Oleic acid (18:1n9)**	R	**0.228**	0.115	**0.150**	**0.214**	**0.205**	**0.246**	−**0.390**	**0.171**	**0.604**
	P	**0.001**	0.090	**0.026**	**0.001**	**0.002**	**0.023**	**<0.001**	**0.011**	**<0.001**
*Saturated FAs*										
**Myristic acid (14:0)**	R	0.130	0.030	0.043	**0.161**	0.057	0.154	−**0.210**	**0.143**	**0.407**
	P	0.053	0.661	0.523	**0.017**	0.405	0.158	**0.002**	**0.034**	**<0.001**
**Palmitic acid (16:0)**	R	0.128	0.089	0.057	**0.138**	0.047	0.116	−**0.149**	−0.041	**0.435**
	P	0.058	0.186	0.397	**0.042**	0.493	0.290	**0.027**	0.541	**<0.001**
**Stearic acid (18:0)**	R	−0.093	−0.075	−0.050	−0.123	−0.099	−0.111	**0.266**	−**0.245**	−**0.379**
	P	0.167	0.270	0.461	0.069	0.144	0.313	**<0.001**	**<0.001**	**<0.001**
*Desaturases*										
**D6D**	R	**0.308**	**0.144**	0.132	**0.262**	**0.262**	**0.347**	−**0.174**	**0.174**	**0.235**
	P	**<0.001**	**0.033**	0.051	**<0.001**	**<0.001**	**0.001**	**0.010**	**0.010**	**<0.001**
**D5D**	R	−**0.195**	−0.037	−0.027	−**0.222**	**−0.176**	−**0.264**	**0.226**	−**0.215**	−**0.209**
	P	**0.004**	0.583	0.695	**0.001**	**0.009**	**0.015**	**0.001**	**0.001**	**0.002**
**SCD**	R	**0.316**	0.120	**0.169**	**0.198**	**0.286**	**0.441**	−**0.168**	**0.245**	**0.455**
	P	**<0.001**	0.075	**0.012**	**0.003**	**<0.001**	**<0.001**	**0.012**	**<0.001**	**<0.001**
**D9D**	R	**0.192**	0.108	0.101	**0.213**	**0.182**	**0.227**	−**0.400**	**0.229**	**0.655**
	P	**0.004**	0.112	0.134	**0.002**	**0.007**	**0.037**	**<0.001**	**0.001**	**<0.001**

AA, arachidonic acid; ALA, α-linolenic acid; BMI, body mass index; BP, blood pressure; D5D, delta-5 desaturase; D6D, delta-6 desaturase; D9D, delta-9 desaturase; DGLA, dihomo-γ-linolenic acid; DPA, docosapentaenoic acid; DHA, docosahexaenoic acid; EPA, eicosapentaenoic acid; GLA, γ-linolenic acid; HDL, high-density lipoprotein; LA, linoleic acid; LDL, low-density lipoprotein; POA, palmitoleic acid; SCD, stearoyl-coenzyme A desaturase; TG, triglycerides.

**Table 5:** Correlations of serum fatty acids (FAs) with glucose and insulin measurements for all women. R = Pearson correlation coefficient. Significant correlations (*p* < 0.05) are displayed in bold.

		Glucose (Fasting)	Insulin (Fasting)	HOMA-IR	HOMA-β-Cell	Glucose AUC	Insulin AUC	HbA1c
		*n* = 220	*n* = 216	*n* = 214	*n* = 214	*n* = 176	*n* = 166	*n* = 220
*ω-3 PUFAs*								
**ALA (18:3n3)**	R	**0.181**	**0.247**	**0.289**	**0.183**	**0.243**	**0.283**	0.111
	P	**0.007**	**<0.001**	**<0.001**	**0.007**	**0.001**	**<0.001**	0.100
**EPA (20:5n3)**	R	0.060	0.029	0.046	−0.030	−0.113	−0.120	0.003
	P	0.374	0.676	0.504	0.665	0.137	0.125	0.969
**DPA (22:5n3)**	R	0.061	−0.121	−0.046	−**0.200**	−0.073	−**0.169**	0.032
	P	0.368	0.077	0.501	**0.003**	0.333	**0.030**	0.634
**DHA (22:6n3)**	R	0.005	−0.126	−0.109	−0.131	−**0.239**	−0.149	−0.059
	P	0.940	0.064	0.113	0.056	**0.001**	0.056	0.382
*ω-6 PUFAs*								
**LA (18:2n6)**	R	−**0.239**	−**0.351**	−**0.395**	−0.093	−**0.346**	−**0.294**	−**0.164**
	P	**<0.001**	**<0.001**	**<0.001**	0.177	**<0.001**	**<0.001**	**0.015**
**GLA (18:3n6)**	R	0.010	0.119	0.105	0.090	0.145	**0.172**	0.108
	P	0.882	0.080	0.127	0.188	0.055	**0.027**	0.110
**DGLA (20:3n6)**	R	−0.132	**0.205**	0.079	**0.222**	0.144	**0.315**	−0.117
	P	0.051	**0.002**	0.250	**0.001**	0.056	**<0.001**	0.083
**AA (20:4n6)**	R	−0.008	−**0.187**	−**0.169**	−**0.196**	−**0.219**	−**0.220**	−0.001
	P	0.904	**0.006**	**0.013**	**0.004**	**0.003**	**0.004**	0.982
*MUFAs*								
**POA (16:1n7)**	R	0.063	**0.286**	**0.256**	0.104	**0.432**	**0.277**	0.054
	P	0.350	**<0.001**	**<0.001**	0.130	**<0.001**	**<0.001**	0.422
**Vaccenic acid (18:1n7)**	R	0.055	0.084	0.108	<0.001	0.129	0.012	−0.024
	P	0.420	0.220	0.114	0.995	0.088	0.878	0.719
**Oleic acid (18:1n9)**	R	**0.201**	**0.297**	**0.331**	0.126	**0.334**	**0.297**	0.125
	P	**0.003**	**<0.001**	**<0.001**	0.066	**<0.001**	**<0.001**	0.064
*Saturated FAs*								
**Myristic acid (14:0)**	R	0.059	**0.253**	**0.256**	**0.168**	**0.298**	**0.304**	0.062
	P	0.380	**<0.001**	**<0.001**	**0.014**	**<0.001**	**<0.001**	0.357
**Palmitic acid (16:0)**	R	**0.175**	**0.239**	**0.292**	0.081	**0.273**	**0.180**	**0.188**
	P	**0.009**	**<0.001**	**<0.001**	0.240	**<0.001**	**0.020**	**0.005**
**Stearic acid (18:0)**	R	−0.117	−0.085	−0.125	−0.050	−**0.176**	−0.086	−0.132
	P	0.084	0.212	0.068	0.465	**0.020**	0.270	0.051
*Desaturases*								
**D6D**	R	−0.016	**0.331**	**0.232**	**0.216**	**0.290**	**0.392**	−0.038
	P	0.818	**<0.001**	**0.001**	**0.002**	**<0.001**	**<0.001**	0.578
**D5D**	R	0.101	−**0.258**	−**0.152**	−**0.262**	−**0.256**	−**0.351**	0.093
	P	0.135	**<0.001**	**0.026**	**<0.001**	**0.001**	**<0.001**	0.167
**SCD**	R	0.011	**0.268**	**0.214**	0.110	**0.411**	**0.279**	−0.004
	P	0.868	**<0.001**	**0.002**	0.107	**<0.001**	**<0.001**	0.949
**D9D**	R	**0.234**	**0.227**	**0.300**	0.083	**0.340**	**0.239**	**0.203**
	P	**<0.001**	**0.001**	**<0.001**	0.227	**<0.001**	**0.002**	**0.003**

AA, arachidonic acid; ALA, α-linolenic acid; AUC, area under the curve; D5D, delta-5 desaturase; D6D, delta-6 desaturase; D9D, delta-9 desaturase; DGLA, dihomo-γ-linolenic acid; DPA, docosapentaenoic acid; DHA, docosahexaenoic acid; EPA, eicosapentaenoic acid; GLA, γ-linolenic acid; HOMA-β-cell, homeostatic model assessment of β-cell function; HOMA-IR, homeostatic model assessment of insulin resistance; LA, linoleic acid; MUFA, monounsaturated fatty acid; POA, palmitoleic acid; PUFA, polyunsaturated fatty acid; SCD, stearoyl-coenzyme A desaturase.

**Table 6:** Characteristics of the subgroup of women at high risk of developing type 2 diabetes (T2D) owing to the requirement for insulin treatment during GDM. This group was divided into women who had or had not developed T2D by 6 years after pregnancy.

	NGT or IGT (*n* = 32)		T2D (*n* = 21)		*P*
	Mean	SD	Mean	SD	
Ethnicity (% Scandinavian)	29		27		0.57
BMI (kg/m^2^)	29.3	4.9	29.1	4.9	0.92
BMI change from pre-pregnancy (kg/m^2^)	−1.27	3.82	−0.41	3.06	0.38
BP Systolic (mmHg)	121	13	120	12	0.72
BP Diastolic (mmHg)	78	9	78	9	0.81
Waist (cm)	96	10	95	14	0.81
Hip (cm)	108	10	107	10	0.82
s-HDL (mM)	1.33	0.33	1.33	0.50	0.96
s-LDL (mM)	3.20	0.66	3.04	0.63	0.38
s-Triglycerides (mM)	1.07	0.34	1.48	1.62	0.18
b-HbA1c (mmol/mol)	40	5	57	23	<0.001
p-Glucose fasting (mM)	5.8	0.4	9.0	4.3	0.001
*Significantly different FAs:*					
Palmitic acid (16:0)	23.0	1.8	24.7	2.2	0.005
LA (18:2n6)	30.4	4.3	27.0	3.9	0.007

b, whole blood; BP, blood pressure; BMI, body mass index; FA, fatty acid; GDM, gestational diabetes mellitus; HDL, high-density lipoprotein; IGT, impaired glucose tolerance; LA, linoleic acid; LDL, low-density lipoprotein; NGT, normal glucose tolerance; p, plasma; s, serum; SD, standard deviation.
